# Homozygous synonymous *FAM111A* variant underlies an autosomal recessive form of Kenny-Caffey syndrome

**DOI:** 10.1038/s10038-024-01301-1

**Published:** 2024-11-06

**Authors:** Loisa Dana Bonde, Ibrahim M. Abdelrazek, Lara Seif, Malik Alawi, Khaled Matrawy, Karim Nabil, Ebtesam Abdalla, Kerstin Kutsche, Frederike Leonie Harms

**Affiliations:** 1 Institute of Human Genetics, University Medical Center Hamburg-Eppendorf, Hamburg, Germany; 2 Department of Human Genetics, Medical Research Institute, Alexandria University, Alexandria, Egypt; 3 Bioinformatics Core, University Medical Center Hamburg-Eppendorf, Hamburg, Germany; 4 Diagnostic Radiology and Medical Imaging Department, Medical Research Institute, Alexandria University, Alexandria, Egypt; 5 Department of Ophthalmology, Faculty of Medicine, Alexandria University, Alexandria, Egypt

**Keywords:** Genetics, Clinical genetics

## Abstract

FAM111A (family with sequence similarity 111 member A) is a serine protease and removes covalent DNA-protein cross-links during DNA replication. Heterozygous gain-of-function variants in *FAM111A* cause skeletal dysplasias, such as the perinatal lethal osteocraniostenosis and the milder Kenny-Caffey syndrome (KCS). We report two siblings born to consanguineous parents with dysmorphic craniofacial features, postnatal growth retardation, ophthalmologic manifestations, hair and nail anomalies, and skeletal abnormalities such as thickened cortex and stenosis of the medullary cavity of the long bones suggestive of KCS. Using exome sequencing, a homozygous synonymous *FAM111A* variant, NM_001312909.2:c.81 G > A; p.Pro27=, that affects the last base of the exon and is predicted to alter *FAM111A* pre-mRNA splicing, was identified in both siblings. We identified aberrantly spliced *FAM111A* transcripts, reduced *FAM111A* mRNA levels, and near-complete absence of FAM111A protein in fibroblasts of both patients. After treatment of patient and control fibroblasts with different concentrations of camptothecin that induces covalent DNA-protein cross-links, we observed a tendency towards a reduced proportion of metabolically active cells in patient compared to control fibroblasts. However, under these culture conditions, we did not find consistent and statistically significant differences in cell cycle progression and apoptotic cell death between patient and control cells. Our findings show that FAM111A deficiency underlies an autosomal recessive form of *FAM111A*-related KCS. Based on our results and published data, we hypothesize that loss of FAM111A and FAM111A protease hyperactivity, as observed for gain-of-function patient-variant proteins, may converge on a similar pathomechanism underlying skeletal dysplasias.

## Introduction

FAM111A is a serine protease with an N-terminal proliferating cell nuclear antigen (PCNA) interacting peptide (PIP) box domain, two central ubiquitin-like domains (UBL-1 and UBL-2), the latter of which overlaps with a single-strand DNA binding domain (DBD), and a C-terminal trypsin 2-like serine protease domain (SPD) (Fig. S1) [1, 2]. FAM111A is recruited to cellular DNA replication sites, where it acts as a positive regulator of DNA replication, especially by regulating PCNA loading at the replication fork and promoting S phase entry and DNA synthesis [3–5]. Moreover, FAM111A removes chemically induced covalent DNA-protein crosslinks (DPCs), such as those induced by the topoisomerase 1 (TOP1) inhibitor camptothecin (CPT), in a protease activity-dependent manner at the replication fork. This process prevents DNA replication fork stalling and DNA strand breaks and promotes progression of replication [6]. Loss or excess of FAM111A results in the inhibition of DNA replication and transcription, DNA damage, and induction of caspase-dependent apoptosis in the human osteosarcoma cell line U2OS and the human chronic myelogenous leukemia cell line HAP1 [4, 6, 7].

In humans, proper FAM111A function appears to be critical. Heterozygous *FAM111A* pathogenic variants cause two skeletal dysplasias, the perinatal lethal gracile bone dysplasia (MIM 602361), also known as osteocraniostenosis, and the clinically milder Kenny-Caffey syndrome, type 2 (KCS; MIM 127000), according to the Online Mendelian Inheritance in Man (OMIM) database [8, 9]. The 2023 revision of the Nosology of Genetic Skeletal Disorders [10] introduced a dyadic naming system to reclassify *FAM111A*-related dysplasias as “osteocraniostenosis, *FAM111A*‐related” (NOS 21‐0060) and “Kenny‐Caffey syndrome, dominant, *FAM111A*‐related” (NOS 21‐0050). This renaming helps to avoid confusion with the clinically similar “Sanjad‐Sakati syndrome, recessive, *TBCE*‐related” (NOS 21‐0040) and to reflect the phenotype originally described by Kenny and Linarelli and Caffey [10–12].

*FAM111A*-related KCS and osteocraniostenosis are characterized by intrauterine growth retardation and/or postnatal short stature, thickened cortex and stenosis of the medullary cavity of long bones, and primary hypoparathyroidism with hypocalcemia and hyperphosphatemia. Additional clinical features of patients with *FAM111A*-associated KCS are relative macrocephaly, facial dysmorphism, and ophthalmologic and dental abnormalities (Table 1). In contrast, patients with osteocraniostenosis have microcephaly and a cloverleaf-shaped skull, decreased skull ossification, micromelia, and thin ribs with thoracic and pulmonary hypoplasia leading to respiratory insufficiency and early death [8].

**Table 1:** Comparison of the clinical features between the siblings with the homozygous synonymous *FAM111A* variant and patients with *FAM111A*-related Kenny-Caffey syndrome reported in the literature

	Family in this study		*FAM111A*-related Kenny-Caffey syndrome
	Patient 1	Patient 2	46 patients from 38 families reported in the literature
General information			
*FAM111A* variant (NM_001312909.2)	c.81 G > A (homozygous), r.[81 g > a, -37_81del, 50_81del]		heterozygous missense and *in-frame* deletion variants (see Fig. S1)
Prenatal findings	NA	NA	intrauterine growth retardation (7/24)
Craniofacial abnormalities			
Abnormality of the face	triangular face, prominent forehead, high anterior hairline, underdeveloped supraorbital ridges	triangular face, prominent forehead, high anterior hairline	triangular face (12/18), prominent forehead (26/29), narrow palpebral fissures (13/20), midface retrusion (8/19)
Abnormal ear morphology	low-set, posteriorly rotated ears, small earlobes, prominent antihelix	low-set, posteriorly rotated ears, prominent antihelix	low-set ears (9/20)
Abnormal eye morphology	deeply set eyes, narrow palpebral fissures, bilateral microphthalmos, blue sclerae, hypotelorism, abnormal optic disc morphology, hypopigmentation of the fundus, dilatation of large choroidal vessels	deeply set eyes, bilateral microphthalmos, blue sclerae, hypotelorism, abnormal optic disc morphology, hypopigmentation of the fundus, dilatation of large choroidal vessels	deeply set eyes (7/8), microphthalmia (4/22), papilledema/pseudopapilledema (4/21)
Abnormal nasal morphology	prominent nasal bridge, long and narrow nose, low hanging columella, underdeveloped nasal alae	narrow nose	short nose and narrow nasal ridge (5/15)
Abnormal oral morphology	short philtrum, thick lower lip vermilion	thick lower lip vermilion, downturned corners of mouth	thin upper lip vermilion (5/8)
Abnormality of the dentition	microdontia of primary teeth	─	enamel hypoplasia, microdontia, hypodontia/oligodontia and/or abnormality of dental eruption (17/22), carious teeth (9/17), premature loss of secondary dentition (4/14)
Abnormal eye and ear physiology			
Abnormality of refraction	high hypermetropia, astigmatism, fully accommodative esotropia, strabismus	high hypermetropia, astigmatism, fully accommodative esotropia	hypermetropia or myopia and/or astigmatism (29/35)
Hearing abnormality	─	─	hearing impairment (4/9)
Abnormal skeletal morphology			
Abnormality of body height	short stature	short stature	short stature/postnatal growth retardation (40/43)
Abnormality of skull size	relative macrocephaly	─	microcephaly (1 patient), relative macrocephaly (7 patients)
Abnormal skull morphology	dolichocephaly, thickened calvaria	dolichocephaly	craniosynostosis (4/21), wide anterior fontanel/delayed closure of the anterior fontanel (26/32), decreased skull ossification (3/11), micrognathia or microretrognathia (15/21), mandibular prognathia (1 patient)
Abnormal axial skeleton morphology	pectus carinatum, thoracic scoliosis	─	scoliosis (3 patients), thin ribs (1/21)
Subperiosteal bone formation	yes	yes	─
Abnormal long bone morphology	thickened cortex of long bones, stenosis of the medullary cavity of the long bones, slender long bones	thickened cortex of long bones, stenosis of the medullary cavity of the long bones	cortical thickening of long bones (31/36), stenosis of medullary cavity of long bones (32/36), slender long bones (6/24)
Abnormal limb bone morphology	─	─	acromicria (3/22)
Abnormality of skeletal maturation	delayed skeletal maturation	delayed skeletal maturation	delayed skeletal maturation (4 patients)
Miscellaneous			
Neurodevelopmental abnormalities	─	─	intellectual disability (5/19)
Abnormality of the integument	thin skin, sparse scalp hair, sparse eyebrows, nail dysplasia	hypermelanotic macule, sparse scalp hair, sparse eyebrows, nail dysplasia	sparse scalp hair (6 patients), nail dysplasia (2 patients)
Abnormality of the voice	high-pitched voice, hypernasal speech	high-pitched voice, nasal speech	high-pitched voice (2 patients)
Abnormality of the respiratory system	pulmonary hypoplasia	─	respiratory distress (1 patient)
Abnormality of the genital system	─	micropenis, cryptorchidism	micropenis (4 patients), decreased testicular size (5 patients)
Abnormality of blood and blood-forming tissues	anemia, thrombocytosis	anemia, thrombocytosis	anemia (1 patient)
Abnormality of the endocrine system and of circulating metabolite concentration	reduced circulating growth hormone concentration, decreased response to growth hormone stimulation test	─	primary hypoparathyroidism with hypocalcemia and/or hyperphosphatemia (40/46), hypomagnesemia (14/21), hypokalemia (4/6), reduced circulating growth hormone concentration (5 patients)
Other abnormalities	recurrent infections, prominent superficial veins, spina bifida occulta at L5	spina bifida occulta at L5	recurrent infections (5 patients), chronic kidney disease (6/9), other renal insufficiency (3 patients)

*─* feature absent, *NA* not available

To date, 46 patients from 38 families with KCS and 14 unrelated patients with osteocraniostenosis and a *FAM111A* pathogenic variant have been reported in the literature [9, 13–38]. The majority of *FAM111A* variants occurred de novo, with the exception of two confirmed cases of maternal and two of paternal inheritance [15, 25, 36, 37]. One patient had compound heterozygous *FAM111A* variants and was the first with an autosomal recessive form of osteocraniostenosis [27]. With the exception of two *in-frame* deletions, all other *FAM111A* pathogenic variants are missense variants [9, 13–38]. The variants cluster in a flexible hinge region between the UBL-2 domain and the SPD, the SPD itself, and the C-terminal region of FAM111A (Fig. S1). Functional studies of selected FAM111A patient-variant proteins in heterologous cell systems revealed increased intrinsic protease activity, suggesting a gain-of-function mechanism of KCS- and osteocraniostenosis-associated *FAM111A* variants [4, 6].

Here we report two siblings with clinical features of KCS carrying a homozygous synonymous c.81 G > A; p.Pro27= variant in *FAM111A*. The variant affects the splicing of *FAM111A* pre-mRNAs leading to almost complete absence of FAM111A protein in patient-derived fibroblasts. Our data suggest that FAM111A deficiency causes an autosomal recessive form of *FAM111A*-related KCS.

## Material and methods

### Study approval

**Fig. 1:**
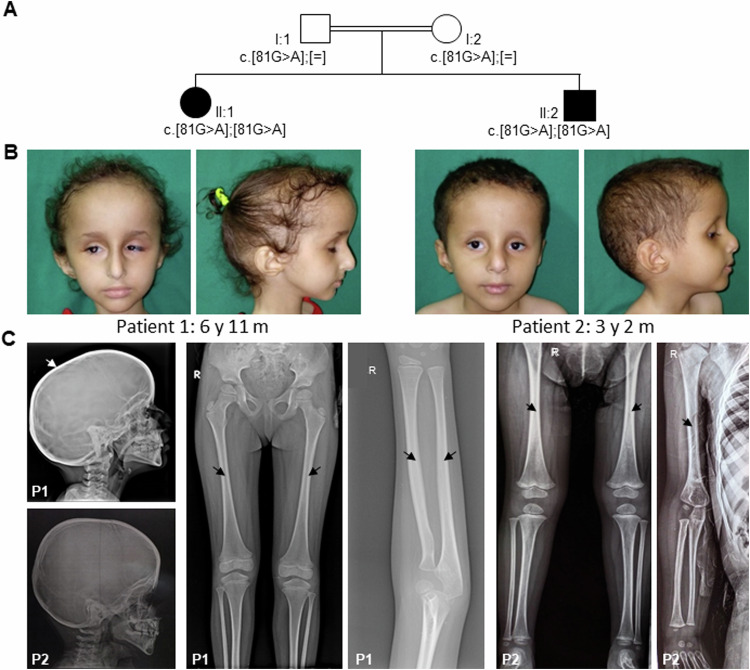
Pedigree, facial photographs, and radiographs of both affected siblings with the homozygous *FAM111A* variant c.81 G > A. **A** Pedigree of the patients’ family. The healthy father (I:1) and the healthy mother (I:2) are first-degree cousins and heterozygous carriers of the *FAM111A* c.81 G > A variant. Both affected siblings, patient 1 (II:1) and patient 2 (II:2), carry the *FAM111A* c.81 G > A variant in the homozygous state. **B** Facial photographs of patient 1 at the age of 6 years and 11 months (left) and of patient 2 at the age of 3 years and 2 months (right) show prominent forehead, triangular face, sparse eyebrows, deeply set eyes, bilateral microphthalmia, a long and narrow nose with a prominent nasal bridge, and posteriorly rotated ears with a prominent antihelix. **C** Radiographs of patient 1 (P1) at the age of 6 years and 10 months and of patient 2 (P2) at the age of 2 years (right upper limb) and 3 years and 2 months (skull and lower limbs). Skull radiographs show mild cortical thickening of the skull with narrowed diploic space indicated by a white arrow. Upper and lower limb radiographs show thickened cortex of long bones and stenosis of the medullary cavity of the long bones in both patients indicated by black arrows. m months, y years

Informed consent for genetic analyses was obtained for both patients, and genetic studies were performed as approved by the Ethics Committee of the Hamburg Medical Chamber (PV7038-4438-BO-ff; Hamburg, Germany). The parents of the affected individuals provided written informed consent for participation in the study, clinical data and specimen collection, and publication of relevant findings, including photographs and x-rays shown in Figs. 1B, C.

### Exome sequencing and variant validation and segregation

Exome sequencing (ES) was performed on genomic DNA extracted from leukocytes of patients 1 and 2 by CeGaT (Tuebingen, Germany). The *FAM111A* variant was validated and segregated by Sanger sequencing using fibroblast-derived DNA (patients 1 and 2) and leukocyte-derived DNA (patients 1 and 2 and parents). For details see Supplementary information and Table S1. Primer sequences can be found in Table S2.

### Cell culture

Primary dermal fibroblasts were cultured from skin biopsies of patients 1 and 2, one healthy male (Ctrl. 1, 9 years old), and three female healthy controls (Ctrl. 2–4, 4 years old) in Dulbecco’s modified Eagle medium (DMEM; Thermo Fisher Scientific, Waltham, MA, USA) supplemented with 10% fetal bovine serum (FBS; GE Healthcare, Chicago, IL, USA) and penicillin-streptomycin (100 U/mL and 100 mg/mL, respectively; Thermo Fisher Scientific). The same passage number of patient and control fibroblasts was used in all experiments. Primary fibroblasts were regularly tested for mycoplasma contamination and confirmed to be mycoplasma-free. To inhibit nonsense-mediated mRNA decay, fibroblasts were cultured in 10 µg/mL cycloheximide-containing DMEM for 6 h prior RNA isolation.

### RNA isolation, cDNA synthesis, RT-PCR and Sanger sequencing, and real-time quantitative PCR (RT-qPCR)

RNA isolation from fibroblasts, complementary DNA (cDNA) synthesis, reverse transcription polymerase chain reaction (RT-PCR), and direct Sanger sequencing of amplicons were performed as previously described (Supplementary information) [39]. Selected PCR products were cloned into the pCR2.1 TOPO TA cloning vector (Thermo Fisher Scientific). Individual *Escherichia coli* clones were subjected to colony PCR using vector specific primers (M13rev and T7cDNA in Table S2) followed by Sanger sequencing. RT-qPCR was performed to determine the relative mRNA levels of *FAM111A* as previously described (Supplementary information) [39]. 2^-ΔCt^ values were calculated individually for each experiment and expressed as a fold change to control 3. Primer sequences can be found in Table S2.

### Immunoblotting

Whole-cell lysates from patient and control fibroblasts were prepared and immunoblotting was performed as described (Supplementary information) [39]. The antibodies used are described in Supplementary information.

### Cell viability assay

Measurement of metabolically active cells was performed as previously described [39]. Briefly, patient and control fibroblasts were seeded in triplicate in 96-well plates at a density of 1250 cells/well and treated with 0.75 µM, 1 µM, 2.5 µM, or 5 µM camptothecin (CPT; MedChemExpress, Monmouth Junction, NJ, USA) or an equal volume of DMSO for 72 h. The medium was replaced after the first 48 h of incubation. The proportion of metabolically active cells was determined using the cell proliferation reagent WST-1 (Roche, Basel, Switzerland) following the manufacturer’s instructions. Formazan dye produced by metabolically active cells was quantified by measuring the absorbance at 440 nm and 605 nm (reference wavelength) using the Synergy H1 plate reader (Biotek Instruments, Winooski, VT, USA) and the Gen5 v2.09 software (Biotek Instruments).

### Data analysis and statistics

Quantitative data are presented by Prism 8 software (GraphPad Software, Boston, MA, USA) as the mean ± standard deviation (SD). For quantification, one- or two-way ANOVA followed by a Dunnett’s *post hoc* test for multiple comparisons was performed. A *p* ≤ 0.05 was considered statistically significant (*p ≤ 0.05; **p ≤ 0.01; ***p ≤ 0.001; ****p ≤ 0.0001).

## Results

### Clinical reports

Patient 1, a 6-year and 11-month-old girl, was the first-born child (II:1) of consanguineous healthy parents (Fig. 1A). Her younger brother was similarly affected (see below). Clinical features of the siblings are summarized in Table 1. Pregnancy and delivery of patient 1 were uneventful. She was delivered at full term by Caesarian section and weighed 2400 g (-1.99 z).

On examination at the age of 6 years and 11 months, patient 1 displayed a distinct facial gestalt (Fig. 1B), which gradually became more apparent with time. She showed growth retardation with an occipitofrontal head circumference (OFC) of 47.5 cm (–3.5 z), length of 94.5 cm (–5.35 z), and weight of 10 kg (–8.05 z). Her development was normal. She had a high-pitched nasal voice, thin skin, prominent superficial veins, dysplastic finger- and toenails (Fig. S2A), and pectus carinatum.

At the age of 3 years, left lung hypoplasia was identified on chest CT; brain MRI and abdominal and pelvic ultrasonography revealed normal findings. She has been on growth hormone therapy since the age of 3 years. Laboratory tests at the age of 6 years identified anemia and thrombocytosis (Table S3). She was successfully treated for anemia with lactoferrin. Parathyroid hormone and calcitonin levels were within the normal range (Table S3). Ophthalmological assessment revealed bilateral high hypermetropic astigmatism with fully accommodative esotropia. Lenstar biometry and B scan ultrasound identified bilateral microphthalmia. Fundus examination showed bilateral pseudopapilledema, hypopigmentation of the fundus, and dilatation of large choroidal vessels (Fig. S2B). Radiographic skeletal survey showed thickened calvaria, mild subperiosteal bone formation of the right middle ribs, and stenosis of the medullary cavity of the long bones, including both clavicles, humeri, radius, femurs, fibulae, and, to a lesser extent, both tibias (Fig. 1C, Fig. S3). X-ray of the spine revealed left-sided thoracic scoliosis and spina bifida occulta at L5 (Fig. S3).

The 3-year and 7-month-old patient 2 was the younger brother of patient 1 (II:2 in Fig. 1A). He was born full term after an uneventful pregnancy with a birth weight of 2500 g (–1.9 z). Like his sister, he had a high-pitched nasal voice. His motor and language development was appropriate for age.

Upon examination at the age of 3 years and 2 months, he exhibited dysmorphic features similar to his sister (Fig. 1B). He also showed growth retardation at this age: OFC was 47.5 cm (–2.61 z), height was 83.5 cm (–3.56 z), and weight was 10 kg (–3.18 z). He had three hyperpigmented macules on the back and thighs and micropenis with bilateral cryptorchidism. As his sister, he had anemia and thrombocytosis (Table S3), nail dysplasia (Fig. S2A), bilateral high hypermetropic astigmatism with fully accommodative esotropia, bilateral microphthalmia, bilateral pseudopapilledema, hypopigmentation of the fundus, and dilatation of large choroidal vessels (Fig. S2B). Skeletal survey revealed mild subperiosteal bone formation of the right middle and left lower ribs, spina bifida occulta at L5, and thickened cortex. He also had stenosis of the medullary cavity of the long bones, including the outer portions of both clavicles, both femurs, and to a greater extent than their right counterparts, the left humerus, radial, and ulnar bones (Fig. 1C). The anemia was improving with lactoferrin.

### Phenotype-driven analysis by the online tool Phenomizer

We first used the Phenomizer online tool [40, 41], which measures phenotypic similarity based on Human Phenotype Ontology (HPO) terms between query terms and known genetic disorders to identify a possible clinical diagnosis in both affected siblings. The following HPO terms were used: short stature, thickened cortex of long bones, and stenosis of the medullary cavity of the long bones. An association of the clinical features with *FAM111A*-related KCS was the only significant finding (Table S4). Moreover, other clinical features in the affected siblings overlap with the KCS-related clinical spectrum. These include characteristic facial features such as a prominent forehead with relative macrocephaly, triangular face, deeply set eyes, low-set ears, and narrow nose, ectodermal abnormalities, including sparse hair and nail dysplasia, a high-pitched voice, and eye abnormalities, such as hypermetropia and astigmatism (Table 1). Additional clinical features, including microphthalmia and anemia in both siblings, growth hormone deficiency and microdontia of primary teeth in patient 1, and genitourinary anomalies in patient 2 are variable features in patients with KCS (Table 1). Thus, the combination of clinical features in both affected siblings, including the skeletal-specific KCS findings, suggested the clinical diagnosis KCS.

### Molecular genetic investigations

**Fig. 2:**
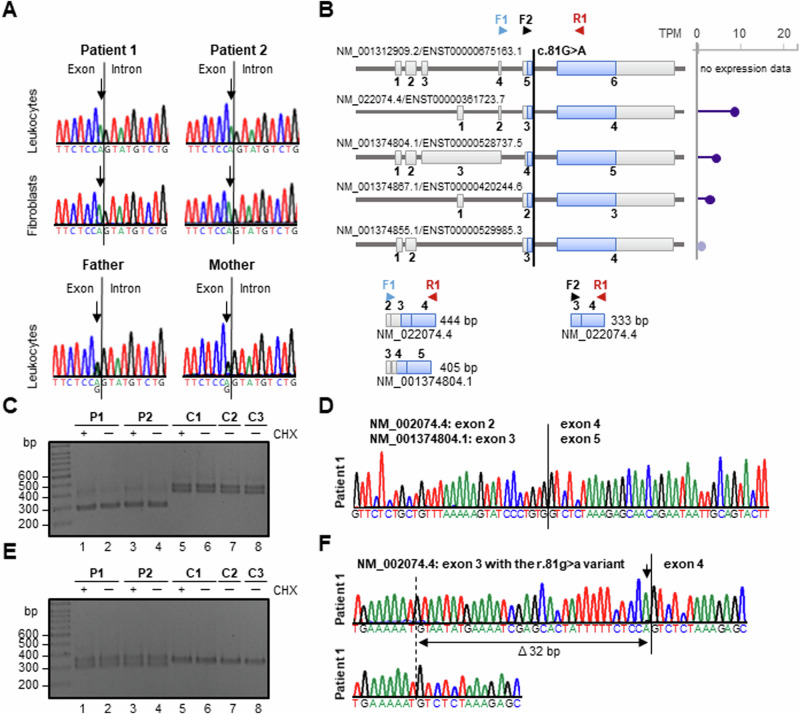
Segregation analysis of the *FAM111A* c.81 G > A variant in the family, *FAM111A* transcript variants, and *FAM111A* transcript analysis in patient and control fibroblasts. **A** Partial sequence electropherograms demonstrating the *FAM111A* NM_001312909.2:c.81 G > A variant in the homozygous state in leukocyte- and fibroblast-derived DNA of patients 1 and 2, and in the heterozygous state in leukocyte-derived DNA of healthy parents (mother and father). The exon-intron boundary is indicated by a black line. Arrows point to the G-to-A change at the last exon position. **B** The MANE *FAM111A* transcript (top) and the four *FAM111A* transcript variants (TVs) expressed in cultured fibroblasts are shown, all encoding the same FAM111A protein. Exons are given by boxes and are numbered. The coding region is indicated in blue, untranslated regions in gray. Expression levels of *FAM111A* TVs in cultured fibroblasts according to the GTEx database (last accessed 08/2024) are shown on the right. Length of the bars represents the rate of expression (violet, strong expression; gray, no expression). Primers used for RT-PCRs are indicated by arrows above the transcript variants. The expected PCR amplicon sizes using the primer combination F1 and R1 (444 bp and 405 bp) and F2 and R1 (333 bp) are depicted in the lower left and the lower right panel, respectively. **C** 2% agarose gel showing RT-PCR amplicons generated with primers F1 and R1 using fibroblast-derived cDNA from patients (P1, P2) and three controls (C1-C3). Fibroblasts were either treated with cycloheximide (CHX, +) or DMSO (─) prior to RNA isolation. In control cells, the expected RT-PCR products of 444 bp and 405 bp were amplified. In contrast, a major amplicon of ~300 bp was obtained from cDNA of patient-derived cells. **D** Partial sequence electropherogram of an aberrantly spliced *FAM111A* transcript in patient 1. Direct sequencing of the 287-bp RT-PCR amplicon obtained with primers F1 and R1 revealed skipping of the r.81 g > a change containing exon 3 (NM_022074.4) or exon 4 (NM_001374804.1) in *FAM111A* transcripts. **E** 2% agarose gel showing RT-PCR amplicons generated with primers F2 and R1 using fibroblast-derived cDNA from patients (P1, P2) and three controls (C1-C3). Fibroblasts were either treated with CHX (+) or DMSO (─) prior to RNA isolation. The expected RT-PCR product of 333 bp was amplified from cDNA of control and patient cells. A second amplicon of ~300 bp was obtained from patient-derived cDNAs. **F** Cloning of patient 1-derived RT-PCR amplicons followed by colony PCR and Sanger sequencing of individual amplicons identified the larger amplicon (333 bp) as transcripts in which exon 3 with the r.81 g > a variant (indicated by an arrow) was correctly spliced to exon 4 (upper electropherogram). The smaller amplicon (301 bp) corresponds to aberrantly spliced *FAM111A* transcripts lacking the last 32 bp of exon 3 (Δ32 bp; lower electropherogram). bp base pairs, F forward primer, R reverse primer, TPM transcripts per million

Duo ES in patients 1 and 2 identified a shared homozygous synonymous variant c.81 G > A; p.Pro27= (MANE select transcript NM_001312909.2) in *FAM111A*, the disease gene for autosomal dominant KCS (Table S1). We did not detect any other rare biallelic variant of pathogenic relevance in an autosomal recessive disease gene that was shared by both siblings and could underlie their phenotype (Table S1). The *FAM111A* c.81 G > A transition was found in 14 out of 1 605 748 alleles and had a worldwide allele frequency of 0.00087% in the gnomAD database v4.1.0 (Table S1) [42] and of 0.001% in the Regeneron Genetics Center Million Exome data [43]. The homozygous variant was validated in leukocyte- and fibroblast-derived DNA from both patients by Sanger sequencing (Fig. 2A). Segregation analysis of the *FAM111A* variant in the healthy parents identified both as heterozygous carriers (Fig. 2A). To predict the effect of the silent c.81 G > A variant, that affects the last base of exon 5 (Fig. 2A, B), we used several in silico splice site prediction programs. All programs predicted a reduced or no recognition of the canonical splice donor site (SDS) in intron 5 (Tables S1, S5). SpliceVault [44] predicted exon 5 skipping as the most likely event.

Based on (i) the low allele frequency of the *FAM111A* variant in the general population, (ii) the predicted splicing alterations, and (iii) the likely clinical diagnosis of KCS in both patients, we believed the homozygous *FAM111A* variant c.81 G > A could underlie the phenotype in the two affected siblings. This prompted us to investigate the effect of the variant on *FAM111A* pre-mRNA splicing and protein levels in patient-derived fibroblasts.

### *FAM111A* transcript analysis

The NCBI database (https://www.ncbi.nlm.nih.gov/gene/63901) annotates 33 *FAM111A* transcript variants (TVs), which differ in the length and exon composition of the 5’ untranslated region (last accessed 08/2024). The *FAM111A* coding region is the same in all TVs, meaning that all encode the same protein composed of 611 amino acid residues. According to the GTEx portal, only four TVs are expressed in primary dermal fibroblasts: NM_022074.4 (ENST00000361723.7), NM_001374804.1 (ENST00000528737.5), NM_001374867.1 (ENST00000420244.6), and NM_001374855.1 (ENST00000529985.3) (Fig. 2B). Expression data for the MANE select transcript NM_001312909.2 (ENST00000675163.1) are not available in the GTEx portal (Fig. 2B).

To analyze the effect of the c.81 G > A variant on splicing of *FAM111A* pre-mRNAs, we performed *FAM111A* transcript analysis using fibroblast-derived cDNA from both patients and controls. We first used a forward primer located in exon 2 (NM_022074.4) or exon 3 (NM_001374804.1) and a reverse primer located in the subsequent exon 4 or 5 (F1 and R1 in Fig. 2B, lower left panel). Two different RT-PCR products were expected in controls due to the different size of exons 3 and 4 in the TVs NM_022074.4 and NM_001374804.1, respectively (Fig. 2B, lower left panel). Two amplicons of the expected size of 444 bp and 405 bp were amplified in controls (Fig. 2C, lanes 6-8). Cloning of control-derived amplicons followed by colony PCR and Sanger sequencing revealed the wild-type sequence of the two TVs (Fig. S4A). In patient-derived fibroblasts, only a single amplicon of ~300 bp was generated by RT-PCR (Fig. 2C, lanes 2 and 4). Direct sequencing of patient-derived amplicons revealed that exon 2 was directly spliced to exon 4 in TV NM_022074.4 (according to NM_001374804.1, exon 3 was spliced to exon 5) (Fig. 2D, Fig. S4B). Skipping of exon 3 in TV NM_022074.4 resulted in loss of 157 bp (r.-76_81del) and of exon 4 in NM_001374804.1 in loss of 118 bp (r.-37_81del), including the ATG start codon and the first 81 bp of the coding region. Treatment of patient and control fibroblasts with the nonsense-mediated mRNA decay (NMD) inhibitor cycloheximide (CHX) prior to RNA isolation and RT-PCR resulted in the same RT-PCR band pattern in control and patient cells compared to untreated cells (Fig. 2C, lanes 1, 3 and 5). The data suggest that no additional aberrantly spliced *FAM111A* transcripts are expressed in patient-derived fibroblasts, which are subjected to NMD.

To specifically amplify *FAM111A* TVs including the exon with the r.81 g > a variant, we used the forward primer F2 located in exon 3 (according to NM_022074.4) and the reverse primer R1 in RT-PCRs (Fig. 2B, lower right panel). Theoretically, all four TVs expressed in fibroblasts could be amplified with this primer combination (Fig. 2B, upper panel). In controls, we obtained an amplicon with the expected size of 333 bp (Fig. 2E, lanes 6-8). Direct sequencing of control-derived amplicons confirmed canonical splicing of exon 3 to exon 4 (Fig. S4C). In patient-derived fibroblasts, we amplified the 333-bp RT-PCR product and a smaller product of ~300 bp (Fig. 2E, lanes 2 and 4). Cloning of patient-derived amplicons followed by colony PCR and Sanger sequencing revealed canonical splicing of exon 3 with the r.81 g > a variant to exon 4 in the larger amplicon (Fig. 2F, upper panel, Fig. S4D, upper panel). In the smaller amplicon, an aberrantly spliced *FAM111A* mRNA lacking the last 32 bp of exon 3 was detected (r.50_81del) (Fig. 2F, lower panel, Fig. S4D, lower panel). Loss of 32 nucleotides in *FAM111A* mRNAs is consistent with the usage of a cryptic SDS in exon 3, located 32 bp upstream of the canonical SDS. This results in a reading frame shift and the introduction of a premature stop codon [p.(Asn18Leufs*2)]. CHX treatment of fibroblasts followed by RNA isolation and RT-PCR resulted in the same band pattern in patient and control cells compared to untreated cells (Fig. 2E, lanes 1, 3 and 5).

In conclusion, the synonymous c.81 G > A variant causes aberrant splicing of *FAM111A* pre-mRNAs in patient-derived fibroblasts, with skipping of exon 3 (according to NM_022074.4) as the likely major event. Nevertheless, canonically spliced *FAM111A* mRNAs with the r.81 g > a variant also exist.

### Determination of relative *FAM111A* transcript and protein levels

**Fig. 3:**
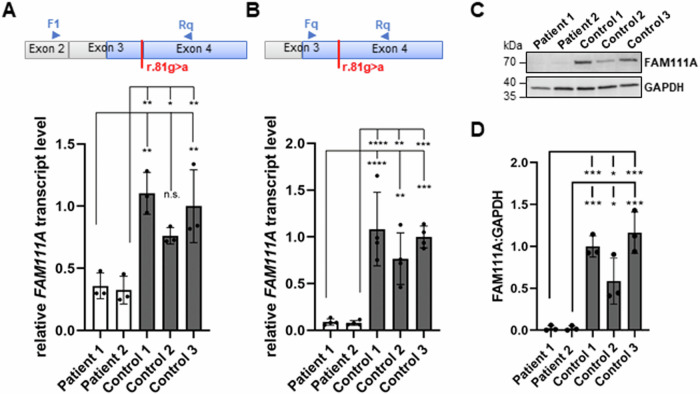
Determination of *FAM111A* transcript and FAM111A protein levels in patient and control fibroblasts. **A**, **B** Relative quantification of *FAM111A* mRNA levels by RT-qPCR using fibroblast-derived cDNA from the two patients and three healthy controls. The schematics above the RT-qPCR data show the location of the F1 primer in exon 2 (**A**), the Fq primer in exon 3 (**B**), and the Rq primer in exon 4 of *FAM111A* (according to NM_022074.4) (**A**, **B**). The coding region is indicated in blue and untranslated region in gray. For quantification, *FAM111A* mRNA levels were normalized to *GAPDH* mRNA levels. The bars and errors represent the mean ± SD of three independent experiments (*n*  =  3), each performed in triplicate. Individual data points are shown. One-way ANOVA followed by Dunnett’s *post hoc* test was used for statistical analysis to compare relative *FAM111A* mRNA levels in fibroblasts from patients 1 and 2 separately with controls 1–3. **C** Representative immunoblot of whole-cell lysates obtained from fibroblasts of patients 1 and 2 and controls 1–3. The amount of FAM111A was monitored by using an anti-FAM111A antibody. Anti-GAPDH antibody was used to control for equal loading. **D** Band intensities of fluorescence signals were quantified using the ChemiDoc imaging system. Levels of FAM111A were normalized to GAPDH. The bars and errors represent the mean ± SD of three independent experiments (*n*  =  3). Individual data points are shown. One-way ANOVA followed by Dunnett’s *post hoc* test was used for statistical analysis to compare the relative FAM111A protein levels in fibroblasts from patients 1 and 2 separately with controls 1–3. *p ≤ 0.05; **p ≤ 0.01; ***p ≤ 0.001; ****p ≤ 0.0001. F1 and Fq forward primers for RT-qPCR, kDa kilodalton, n.s. not significant, Rq reverse primer for RT-qPCR

To determine *FAM111A* mRNA levels, we performed RT-qPCR experiments using a primer combination (F1 and Rq in Fig. 3A, upper panel) that amplifies the two transcripts with the highest expression in cultured fibroblasts (NM_022074.4 and NM_001374804.1 in Fig. 2B). RT-qPCR revealed that *FAM111A* transcript levels were significantly reduced to 33–47% in patient 1 and to 29–43% in patient 2 cells compared to cells of controls 1–3 (Fig. 3A). To determine the proportion of *FAM111A* transcripts containing exon 3 (according to NM_022074.4), we used a forward primer (Fq) located in this exon (Fig. 3B, upper panel). Relative levels of *FAM111A* mRNAs containing the full-length exon 3 with the r.81 g > a change and/or the 3’-shortened exon 3 (r.50_81del) were significantly reduced to 8–12% in patient 1- and to 7–10% in patient 2-derived fibroblasts compared to control fibroblasts (Fig. 3B). These data show that exon skipping is the major aberrant splicing event. In addition, *FAM111A* transcripts with the full-length exon harboring the synonymous r.81 g > a change, which could produce wild-type FAM111A protein, were strongly reduced in patient-derived fibroblasts.

Next, we analyzed FAM111A protein levels in patient- and control-derived fibroblasts. FAM111A was detected at the predicted molecular mass of ~70 kDa in control cells, whereas fibroblasts of both patients contained almost no FAM111A protein (Fig. 3C). Quantitative analysis revealed significantly reduced FAM111A protein levels to 2–4% in patient compared to control fibroblasts (Fig. 3C, D). The data demonstrate that aberrant *FAM111A* pre-mRNA splicing results in markedly decreased FAM111A protein levels in patient cells. Given the residual FAM111A levels, we hypothesize that the c.81 G > A change is a hypomorphic variant.

### Effect of the TOPI inhibitor camptothecin on the proportion of metabolically active cells, cell cycle, and apoptosis

**Fig. 4:**
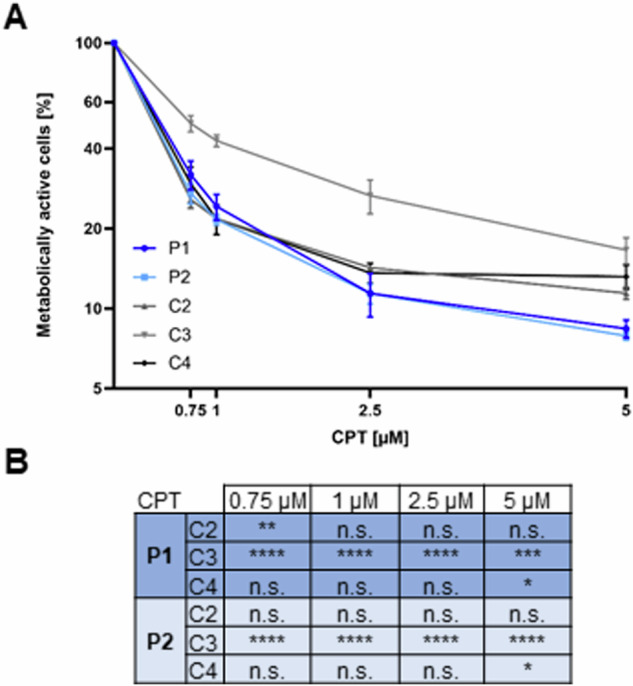
Determination of the proportion of metabolically active cells in patient and control fibroblasts after camptothecin treatment. **A** Patient- and control-derived fibroblasts were treated with camptothecin (CPT) at a concentration of 0.75 µM, 1 µM, 2.5 µM, or 5 µM or with an equal volume of DMSO for 72 h. The graph shows the proportion of metabolically active cells in patient (P1, P2) and control (C2-C4) fibroblasts following treatment with the indicated concentrations of CPT for 72 h relative to DMSO-treated cells. The mean ± SD of three individual experiments is shown (*n* = 3), each performed in triplicate. **B** Two-way ANOVA followed by Dunnett’s *post hoc* test was used for statistical analysis to compare the proportion of metabolically active fibroblasts from patients 1 and 2 separately with that of fibroblasts from controls 2–4. *p ≤ 0.05; **p ≤ 0.01; ***p ≤ 0.001; ****p ≤ 0.0001. n.s. not significant

Chemical induction of DPCs by the TOPI inhibitor CPT decreases cell viability in *FAM111A* knockout HAP1 cells [6]. This observation prompted us to investigate cell viability, that is, to determine the proportion of metabolically active cells, in patient- and control-derived fibroblasts after CPT treatment. Treatment of control fibroblasts with 0.75 µM, 1 µM, 2.5 µM, or 5 µM CPT for 72 h caused a decrease in the proportion of metabolically active cells compared to DMSO-treated cells to ~50% (for 0.75 µM) and to ~17% (for 5 µM) in control 3 cells and to 26–30% (for 0.75 µM) and to 11–13% (for 5 µM) in control 2 and 4 cells (Fig. 4A). In patient-derived fibroblasts, the proportion of metabolically active cells decreased to 32–37% after treatment of cells with 0.75 µM CPT and to ~8% with 5 µM CPT (Fig. 4A). The reduced proportion of metabolically active cells in patient-derived fibroblasts was statistically significant at all CPT concentrations when compared to control 3. Furthermore, reduced proportion of metabolically active cells in patient 1-derived fibroblasts was statistically significant after treatment of cells with 0.75 µM CPT when compared to control 2 and for patient 1- and patient 2-derived fibroblasts after treatment of cells with 5 µM CPT when compared to control 4 (Fig. 4B). Together, the data show that high CPT concentrations have a negative impact on the proportion of metabolically active cells in both patient and control fibroblasts. Our results suggest that patient-derived fibroblasts with minimal FAM111A protein levels were slightly more sensitive to chemically induced DPCs at high CPT concentrations than control fibroblasts.

Treatment of *FAM111A* knockout HAP1 cells with DPC-inducing chemicals leads to cell cycle arrest in the G2/M phase [6]. Therefore, we next performed a cell cycle analysis in fibroblasts of both patients and control 3. In patient and control fibroblasts treated with DMSO, 45–47% of cells were in the G1 phase, 36–42% were in the S phase, and 11–﻿17% were in the G2/M phase (Fig. S5A–C). No statistically significant difference was observed between patient and control fibroblasts (Fig. S5D), suggesting that the almost complete absence of FAM111A in patient fibroblasts does not severely impact the cell cycle under steady-state conditions. Treatment of control fibroblasts with 0.75 µM, 1 µM, 2.5 µM, or 5 µM CPT for 24 h caused an accumulation of almost 100% of cells in the G1 phase of the cell cycle (Fig. S5A), suggesting that high CPT concentrations almost completely block the S phase entry in human fibroblasts. After treatment of cells with 0.05 µM CPT, patient and control fibroblasts accumulated in the S phase (56–70%), with fewer cells in G1 (12–28%; Fig. S5B, C). In contrast, at a concentration of 0.01 µM CPT, we observed fewer cells in the S phase (24–27%) and found an accumulation of both patient and control fibroblasts in the G2/M phase (26–35%; Fig. S5B, C). Together, our analysis did not reveal consistent and significant differences in the distribution of patient and control cells across the analyzed cell cycle phases after treatment with different CPT concentrations (Fig. S5D).

*FAM111A* knockout HAP1 cells show a marked increase in apoptotic cell death after treatment with DPC-inducing chemicals [6]. Therefore, we treated patient and control fibroblasts with 2.5 µM or 5 µM CPT for 72 h and performed an apoptosis assay (Fig. S6A). Cells that were Annexin V-positive and propidium iodide (PI)-negative were considered early apoptotic, while double-positive cells were considered late apoptotic or necrotic [45]. Treatment of cells with 2.5 µM CPT resulted in a 0.8–2.9% increase in the proportion of early apoptotic cells in control cells. In patient 1 cells, we identified an increase of 3.8% and in patient 2 cells an increase of 6.3% in early apoptotic cells (Fig. S6B). The increase in early apoptotic cells was statistically significant for patient 2 cells compared to all controls and for patient 1 cells compared to control 4 (Fig. S6B). We did not detect an increase in late apoptotic cells under this condition (Fig. S6A, B). After treatment of cells with 5 µM CPT, we identified an increase in the proportion of early apoptotic cells of 8.0–21.2% in control cells, of 19.3% in patient 1 cells, and of 37.1% in patient 2 cells (Fig. S6A, B). The increase in early apoptotic cells was statistically significant for patient 2 cells compared to all controls and for patient 1 cells compared to control 4 (Fig. S6B). The percentage of late apoptotic cells increased by 0–2.6% in patient and control fibroblasts with no statistically significant differences between patient and control cell lines (Fig. S6B). Our results show that high CPT concentrations induce cell death in human dermal fibroblasts. However, we did not find a consistent increase in apoptosis in CPT-treated patient compared to control cells. Nevertheless, at least for patient 2 cells, increased apoptosis may be responsible for the observed decrease in the proportion of metabolically active cells (Fig. 4).

## Discussion

Here we report on two siblings with clinical features suggestive of *FAM111A*-related KCS, including relative macrocephaly, characteristic facial features, ophthalmologic manifestations, hair and nail anomalies, proportionate short stature, and highly specific skeletal abnormalities such as thickened cortex and stenosis of the medullary cavity of the long bones. Both siblings shared a homozygous synonymous *FAM111A* variant c.81 G > A; p.Pro27=, which causes aberrant splicing of *FAM111A* pre-mRNAs, including skipping of the first coding exon as the likely major event. FAM111A protein was drastically reduced in patient-derived fibroblasts. Our data suggest that the c.81 G > A variant is a hypomorphic allele leading to FAM111A deficiency and causing an autosomal recessive form of *FAM111A*-related KCS when present in the homozygous state.

The two siblings with KCS and a homozygous silent *FAM111A* variant reported here and the previously described patient with compound heterozygous *FAM111A* variants, c.976 T > A; p.(Leu326Ile) and c.1714_1716del; p.(Ile572del) [27], support the existence of an autosomal recessive form of *FAM111A*-related skeletal dysplasia. The previously reported patient had respiratory distress after birth, facial dysmorphism, microphthalmia, short stature, micromelia, genital abnormalities, hypoparathyroidism with hypocalcemia, and anemia. Skeletal survey revealed decreased skull ossification, short and thin ribs with thoracic hypoplasia, and slender bones. He died at the age of 4.5 months. Based on the severe clinical presentation, he was suggested to have osteocraniostenosis [27]. The *FAM111A* variants p.(Leu326Ile) and p.(Ile572del) are located in the flexible hinge region and the C-terminal end of FAM111A, respectively. Thus, they cluster in the same regions as heterozygous *FAM111A* pathogenic variants (Fig. S1). The p.(Ile572del) variant in *FAM111A* has also been identified in trans with another missense variant, c.881 A > G; p.(Glu294Gly), in one patient from a large cohort of patients with childhood-onset hypoparathyroidism. Additional clinical data for this patient were not available [18]. The amino acid deletion and both missense variants in *FAM111A* have not been functionally characterized. While the p.(Leu326Ile) variant is absent in population databases, the p.(Glu294Gly) variant is rare (allele frequency of 0.000005; gnomAD database v4.1.0). The p.(Ile572del) variant is also rare, but is present in one homozygote (gnomAD database v4.1.0). Thus, occurrence of the p.(Glu294Gly) and p.(Ile572del) variants in the general population suggests that both act as hypomorphic rather than gain-of-function alleles.

*FAM111A* knockdown in the U2OS cancer cell line and in human TIG-3 primary lung fibroblasts reduces the DNA replication rate and cell proliferation [3, 7]. In the HAP1 cancer cell line, *FAM111A* knockout causes cell cycle abnormalities and increased apoptotic cell death after treatment with DPC-inducing chemicals [6]. Given only residual levels of FAM111A in patient fibroblasts with the homozygous *FAM111A* c.81 G > A variant (see Fig. 3C), we assumed similar cellular defects to those found in genetically engineered *FAM111A* knockdown and knockout cell lines. We found, however, that CPT concentrations higher than those used for HAP1 parental and *FAM111A* knockout cells [6] were required to observe negative effects on the viability of fibroblasts from patients and controls. We could not identify consistent alterations in the two patient compared to control cells when examining the cell cycle and apoptotic cell death. These findings could be attributed to cell type-specific functions and/or different expression levels of FAM111A in primary dermal fibroblasts and immortalized cancer cells. Even in cancer cell lines, different cellular effects have been observed upon *FAM111A* knockdown or knockout. *FAM111A* knockdown in U2OS cells resulted in a slight accumulation of cells in the G1 phase under basal culture conditions [7]. In contrast, HAP1 *FAM111A* knockout cells showed no alterations in the distribution of cell cycle phases when treated with DMSO in control experiments [6]. It is possible that other proteases involved in the proteolysis of DPCs, such as the FAM111A homolog FAM111B, the aspartic proteases DDI1 and DDI2, and/or the metalloproteases SPRTN and GCNA (ACRC) [46], could compensate for FAM111A deficiency in primary fibroblast cells.

Most of the *FAM111A* pathogenic variants are present in the heterozygous state in individuals with skeletal dysplasia [9, 13–26, 28–38]. To gain insight into the underlying pathomechanism, functional studies have been performed by ectopic expression of FAM111A wildtype and specific patient-variant proteins in heterologous cell systems, such as HEK293T and U2OS cell lines. FAM111A forms dimers and undergoes autocleavage after Phe334 that separates the SPD from the N-terminal domains [4, 6, 47]. The KCS-related FAM111A-p.(Tyr511His) and -p.(Arg569His), and the osteocraniostenosis-associated FAM111A-p.(Thr338Ala), -p.(Ser343del), and -p.(Asp528Gly) patient-variant proteins exhibit increased autocleavage that can occur intra- and intermolecularly [4, 6]. Interestingly, the protease activity of the isolated FAM111A SPD is ~30-fold higher than that of full-length FAM111A [47]. Together, the data suggest that unrestrained intra- and intermolecular autocleavage of patient-variant FAM111A proteins results in an increased release of hyperactive SPD fragments from the potentially autoinhibitory N-terminus [47]. Thus, hyperactive FAM111A protease activity is the proposed pathomechanism underlying autosomal dominant *FAM111A*-related KCS and osteocraniostenosis and could explain reduced DNA replication, enhanced DNA replication stress and damage, and the induction of apoptotic cell death in cancer cells stably expressing FAM111A patient-variant proteins [4, 7]. Importantly, primary cells derived from patients with heterozygous *FAM111A* gain-of-function variants have not been used as a model system to study the cellular effects of the pathogenic variants. It therefore remains unclear if patient cells show similar cellular defects as cancer cells ectopically expressing patient-variant FAM111A proteins. Of note, overexpression of FAM111A wildtype and FAM111A patient-variant proteins has similar adverse effects on cell fitness as observed in *FAM111A* knockdown or knockout cell lines [4, 7]. Thus, we and others hypothesize that both reduced FAM111A levels and hyperactive FAM111A have similar pathophysiological consequences leading to the development of skeletal dysplasias in humans [1].

In conclusion, we demonstrated that the homozygous synonymous *FAM111A* c.81 G > A variant causes aberrant pre-mRNA splicing and reduced FAM111A protein levels and underlies the KCS phenotype in both siblings reported here. Together with the previously reported osteocraniostenosis-affected boy with compound heterozygous *FAM111A* variants [27], the data show that biallelic *FAM111A* variants can cause autosomal recessive *FAM111A*-related skeletal dysplasias, possibly through a partial loss-of-function mechanism. According to the “Nosology of genetic skeletal disorders: 2023 revision” [10], we suggest to establish a new entity, namely “Kenny‐Caffey syndrome, recessive, *FAM111A*‐related“.

## Supplementary information

Supplemental Material

## Data Availability

The genetic data are not publicly available due to privacy or ethical restrictions. The *FAM111A* variant and phenotypic information reported in this study have been deposited in the Leiden Open Variation Database (http://www.lovd.nl/FAM111A) with the accession IDs #0000989999 (patient 1) and #0000990000 (patient 2).
