# Supplementary material for: Homozygous synonymous FAM111A variant underlies an autosomal recessive form of Kenny-Caffey syndrome
Source: J Hum Genet. 2024 Nov 6;70(2):87–97. doi: 10.1038/s10038-024-01301-1 (PMC11762410; doi:10.1038/s10038-024-01301-1)
Supplement: Supplementary file 1 — Supplemental Material [file 10038_2024_1301_MOESM1_ESM.pdf]

SUPPLEMENTARY INFORMATION

Homozygous synonymous *FAM111A* variant underlies an autosomal recessive form of Kenny-Caffey syndrome

Loisa Dana Bonde[1], Ibrahim M. Abdelrazek[2], Lara Seif[1], Malik Alawi[3], Khaled Matrawy[4], Karim Nabil[5], Ebtesam Abdalla[2], Kerstin Kutsche[1], Frederike Leonie Harms[1]

[1] Institute of Human Genetics, University Medical Center Hamburg-Eppendorf, Hamburg, Germany

[2] Department of Human Genetics, Medical Research Institute, Alexandria University, Alexandria, Egypt

[3] Bioinformatics Core, University Medical Center Hamburg-Eppendorf, Hamburg, Germany

[4] Diagnostic Radiology and Medical Imaging Department, Medical Research Institute, Alexandria University, Alexandria, Egypt

[5] Department of Ophthalmology, Faculty of Medicine, Alexandria University, Alexandria, Egypt

CORRESPONDENCE

Frederike L. Harms, PhD

Institute of Human Genetics

University Medical Center Hamburg-Eppendorf

Martinistraße 52

20246 Hamburg

Germany

Email: fharms@uke.de

SUPPLEMENTARY METHODS

Exome sequencing and variant filtering

Exome sequencing (ES) was performed on genomic DNA extracted from leukocytes of patients 1 and 2 by CeGaT (Tübingen, Germany). Enrichment was carried out using the SureSelect Human All Exon V6 kit (Agilent, Santa Clara, CA, USA). Each captured library was then loaded and sequenced on the HiSeq platform (Illumina, San Diego, CA, USA). fastp (v0.21.0) (1) was used to remove artificial and low quality (Phred quality score below 15) sequences from the 3' end of sequence reads. Putative base calling errors located in regions were two reads of a read pair overlap were corrected (fastp option: '--correction'). The sequences were then aligned to the human reference assembly [NCBI GRCh38 (GCA_000001405.15)] with the Burrows-Wheeler Aligner (BWA mem, v0.7.17-r1188) (2). Strelka2 (v2.9.10) (3) and GATK4 (v4.1.9.0) (4) were used to detect genetic variation. Variants were annotated using the Ensembl Variant Effect Predictor (v103.0) (5). Only biallelic exonic and intronic variants located at exon-intron boundaries ranging from -10 to +10 shared by patients 1 and 2 that were private (absent in the gnomAD database v2.1.1, v3.1.2, and v4.1.0) or rare [with a minor allele frequency (MAF) ≤0.1% and no homozygotes in the gnomAD database v2.1.1, v3.1.2, and v4.1.0] were retained (Table S1). Variants with poor depth of sequencing coverage (total read depth < 10) and in low quality regions (checked in IGV) were discarded.

Variant validation and segregation

Validation of the *FAM111A* variant in leukocyte- and fibroblast-derived DNA of patients 1 and 2 and variant segregation in leukocyte-derived DNA of parents were performed by PCR followed by Sanger sequencing of amplicons. Primers were designed to amplify the variant containing exon of *FAM111A* and adjacent intronic sequences (Table S2). Amplicons were directly sequenced using the ABI BigDye Terminator Sequencing Kit (Applied Biosystems, Waltham, MA, USA) and an automated capillary sequencer (ABI 3500, Applied Biosystems). Sequence electropherograms were analyzed using Chromas v2.6.6 (Technelysium Pty Ltd, South Brisbane, Australia).

RNA isolation and complementary DNA synthesis

Total RNA was extracted from cultured primary fibroblasts of patients 1 and 2 using the Monarch Total RNA Miniprep Kit (New England Biolabs, Frankfurt am Main, Germany). The RNA concentration and purity of the samples were assessed using the Epoch^{TM} Microplate Spectrophotometer (Biotek Instruments, Winooski, VT, USA). 1 µg of total RNA was reverse transcribed using the LunaScript RT Super Mix Kit (New England BioLabs).

Real-time quantitative PCR (RT-qPCR)

Technical triplicates of RT-qPCR samples were prepared as a 10-µL approach using the SYBR Green I-based Luna Universal qPCR Master Mix (New England BioLabs), 500 nM of each primer, and 1 µl of the reverse transcription reaction. Primer sequences for RT-qPCR are described in Table S2. RT-qPCR was performed using the QuantStudio 3 Real-Time PCR System equipped with the QuantStudio Design&Analysis Software v1.4.3 (Thermo Fisher Scientific, Waltham, MA, USA). The PCR conditions consisted of a pre-run at 95°C for 5 minutes, followed by 40 cycles of 30 seconds at 95°C, 30 seconds at 58°C and 45 seconds at 72°C. The specificity of PCR amplification was determined by melting curve analysis with a range from 60°C to 95°C. The cycle threshold (CT) values of the target mRNAs were normalized to the housekeeping mRNA of *GAPDH*.

Immunoblotting

Fibroblasts were collected in ice-cold RIPA buffer [50 mM Tris-HCl, pH 8.0; 150 mM NaCl; 1% NP-40; 0.5% DOC (sodium deoxycholate); 0.1% SDS (sodium dodecyl sulfate)] supplemented with Mini Protease Inhibitor and PhosSTOP (Roche, Basel, Switzerland) and lysed on ice for 10 minutes. Cell debris was removed by centrifugation for 10 minutes. The protein extracts were supplemented with 4x Laemmli buffer and separated on a Mini PROTEAN® TGX Stain-Free Gel (Bio-Rad, Hercules, CA, USA) under denaturing conditions and transferred to polyvinylidene fluoride membranes. Membranes were

blocked in 5% milk in TBST (m-TBST), and then incubated with the monoclonal rabbit anti-FAM111A primary antibody (1:500; #ab184572; Abcam, Cambridge, UK) overnight in blocking solution at 4°C. After washing, StarBright Blue 700-linked anti-rabbit secondary (1:10 000; #12004161; BioRad) and anti-GAPDH hFAB Rhodamine (1:10 000; #12004167; Bio-Rad) antibodies were incubated for 1 hour in 0.5% mTBST at room temperature. Immunoblots were digitally imaged using a ChemiDoc MP (Bio-Rad), with exposure time optimized to avoid saturation. Bands were automatically defined, and intensities were determined using the built-in band detection tool of the Image Lab v6.0 software (Bio-Rad).

Cell cycle analysis

1 x 10^5 patient or control fibroblasts were seeded onto 6-well plates. The following day, fibroblasts were treated with either 0.01 µM, 0.05 µM, 0.75 µM, 1 µM, 2.5 µM or 5 µM camptothecin (CPT) or an equal volume of DMSO for 24 h. After rinsing with PBS, cells were harvested by trypsinization and transferred into flow cytometry tubes. Cells were collected by centrifugation at 600 x g for 5 min, washed once with PBS and fixed with 300 µl 70% ethanol for 30 min at 4°C. After centrifugation, cells were incubated with 50 µl of 100 µg/ml RNAse A/T1 mix (Thermo Fisher Scientific) for 20 min at 37°C following by staining with 100 µl of 50 µg/ml propidium iodide (PI) (Sigma-Aldrich, St. Louis, MO, USA) for 20 min at room temperature in the dark. Fluorescence signals were measured using the FACS NovoCyte Quanteon (Agilent, Santa Clara, CA, USA) equipped with a 598 mirror and 615/20 bandwidth filter with a 488 nm laser and the NovoExpress software v1.6.2 (Agilent).

Apoptosis assay

1.5 x 10^5 patient or control fibroblasts were seeded onto 6-well plates. The following day, the cells were treated with either 2.5 µM or 5 µM CPT or an equal volume of DMSO for 72 h. For the CPT treatment, the medium was replaced after 48 h of incubation. The supernatants were transferred into flow cytometry tubes. Cells were harvested by trypsinization, added to the respective flow cytometry

tube, and washed with PBS by centrifugation at 600 x g for 5 min. The FITC Annexin V Apoptosis Detection Kit I (BD Biosciences, Franklin Lakes, NJ, USA) was used according to manufacturer's instructions to stain for Annexin V and PI, allowing for the identification of early (Annexin V-positive, PI-negative) and late apoptotic/necrotic (Annexin V-positive, PI-positive) cells. The stained cells were analyzed using the BD LSRFortessa flow cytometer (BD Biosciences) equipped with a 488 nm and 561 nm laser and the BD FACSDiva software v.9.0.1 (BD Biosciences). The open source software Flowing Software v2.5.1 (Turku Bioscience, Turku, Finland) was used for gating and analysis. To determine the increase in early and late apoptotic cells that resulted from CPT treatment, the proportion of Annexin V-positive/PI-negative and double-positive cells after DMSO treatment was subtracted from the proportion of Annexin V-positive/PI-negative and double-positive cells after CPT treatment, respectively.

SUPPLEMENTARY FIGURES

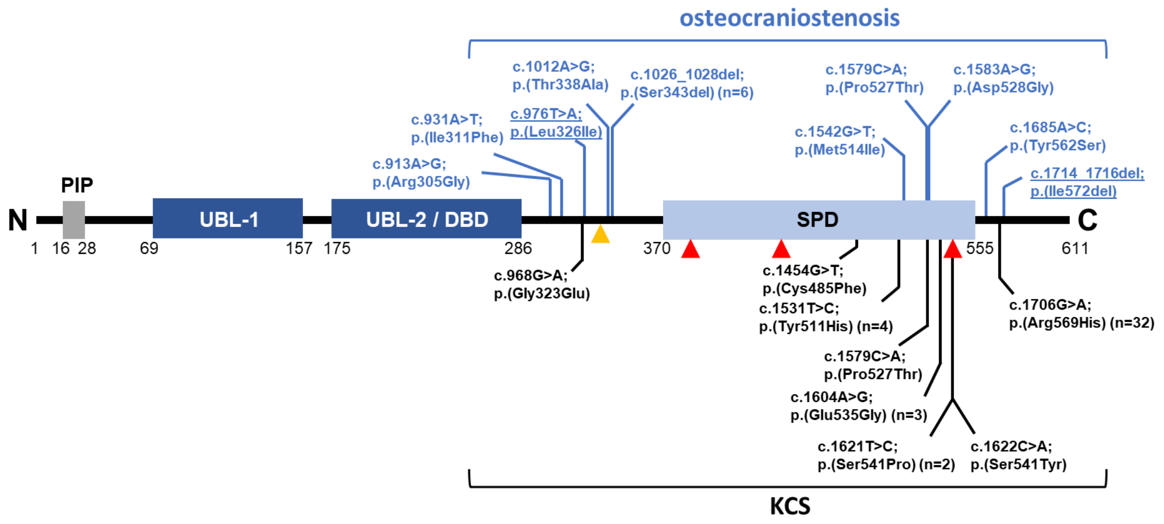

Fig. S1: Schematic representation of the FAM111A domain structure (NP_001299838.1) and location of *FAM111A* pathogenic variants. Amino acid numbering is given. *FAM111A de novo* pathogenic variants associated with Kenny-Caffey syndrome (KCS) and osteocraniostenosis according to the HGMD professional database (last accessed 08/2024) and additional literature are given below (in black) and above the domain structure (in blue), respectively. Note the clustering of pathogenic variants in a flexible hinge region between the UBL-2 domain and the SPD, the SPD itself, and the C-terminal region of FAM111A. Compound heterozygous variants identified in a patient with osteocraniostenosis (6) are underlined. Recurrent variants are indicated by the number of identified patients (n) in brackets. The c.1026_1028del; p.(Ser342del) variant was detected in six unrelated patients, the c.1531T>C; p.(Tyr511His) variant in four patients from three families, the c.1604A>G; p.(Glu535Gly) variant in three patients from one family, the c.1621T>C; p.(Ser541Pro) in monozygotic twins, and the c.1706G>A; p.(Arg569His) variant in 32 patients from 29 families. A yellow arrowhead indicates the autocleavage site at Phe334. Red arrowheads indicate the catalytic triad of His385, Asp439, and Ser541. C, C-terminus; DBD, DNA binding domain; N, N-terminus; PIP, PCNA (proliferating cell nuclear antigen) interacting peptide box; SPD, trypsin 2-like serine protease domain; UBL-1/2, ubiquitin-like domains 1 and 2.

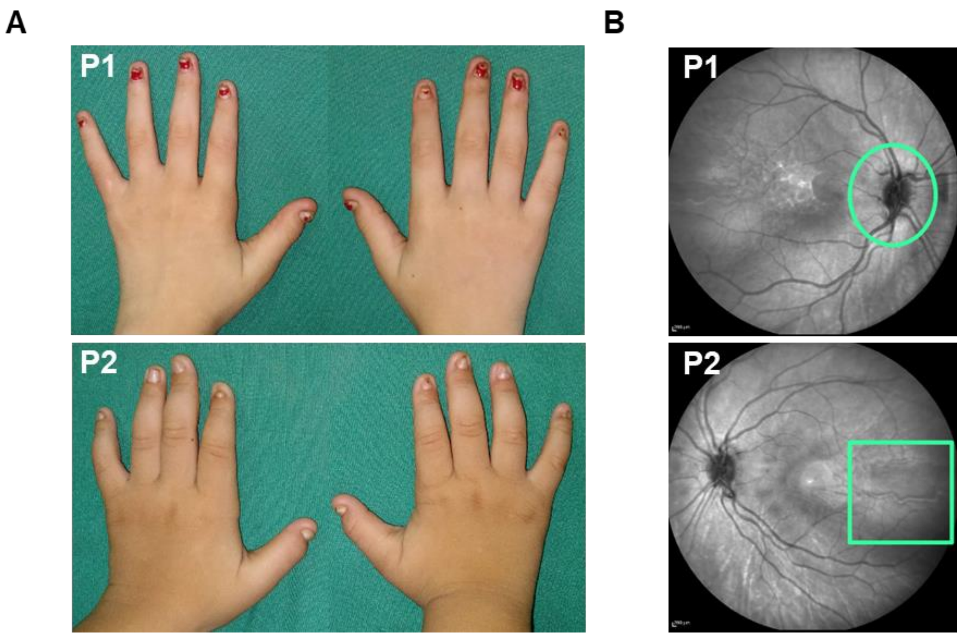

Fig. S2: A Photographs of the hands of patients 1 (P1) and 2 (P2) at the age of 6 years and 11 months (top) and 3 years and 2 months (bottom), respectively, show nail dysplasia. **B** Fundus examination of patient 1 at the age of 6 years and 11 months showed pseudopapilledema (top image; highlighted with a green circle) and hypopigmentation of the fundus. Fundus examination of patient 2 at the age of 3 years and 2 months showed bilateral dilatation of large choroidal vessels (bottom image, highlighted with a green rectangle).

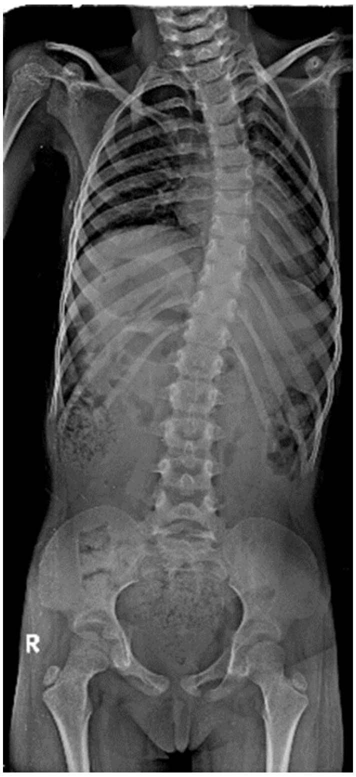

Fig. S3: Posterior-anterior chest and abdominal radiograph of patient 1 at the age of 6 years and 10 months. Patient 1 showed left-sided dorsal scoliosis and thickened cortex and stenosis of the medullary cavity of both clavicles.

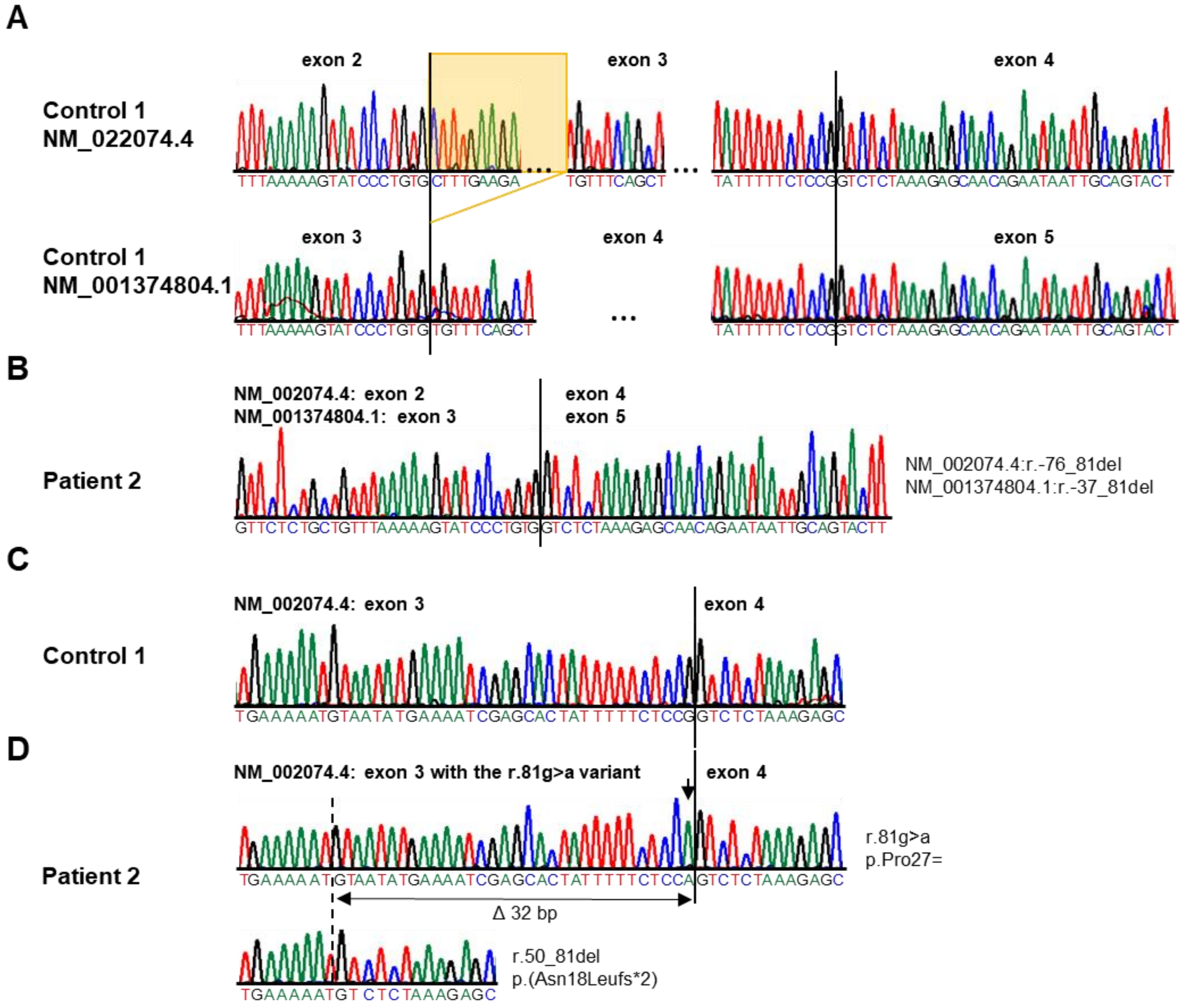

Fig. S4: Additional partial sequence electropherograms of *FAM111A* transcripts in patient 2- and control-derived fibroblasts. Exon-exon boundaries are marked by black lines. **A** Partial sequence electropherograms of wild-type *FAM111A* transcripts identified in control 1 cells. Cloning of control 1-derived RT-PCR amplicons obtained with the primer combination F1 and R1 (**see Fig. 2B, C**) into pCR2.1 TOPO TA cloning vector followed by colony PCR and Sanger sequencing of individual amplicons identified the larger amplicon (444 bp) to represent canonically spliced *FAM111A* (NM_022074.4) transcripts. The smaller amplicon (405 bp) also represents canonically spliced *FAM111A* (NM_001374804.1) transcripts. The sequence unique to exon 3 of NM_022074.4 is highlighted in yellow. **B** Partial sequence electropherogram of the aberrantly spliced *FAM111A* transcripts in patient 2. Direct sequencing of the 287-bp RT-PCR amplicon obtained with primers F1 and R1 (**see Fig. 2B, C**) revealed skipping of exon 3 (NM_022074.4) or exon 4 (NM_001374804.1) harboring the r.81g>a change in *FAM111A* transcripts. **C** Partial sequence electropherogram of the canonically spliced *FAM111A* transcript in control 1 cells obtained from direct Sanger sequencing of the RT-PCR product obtained with primers F2 and R1 (**see Fig. 2B, E**). **D** Cloning of patient 2-derived RT-PCR amplicons followed by colony PCR and Sanger sequencing of individual amplicons identified the larger amplicon (333 bp) as transcripts in which exon 3 with the r.81g>a variant (indicated by an arrow) was correctly spliced to exon 4 (upper electropherogram). The smaller amplicon (301 bp) corresponds to aberrantly spliced *FAM111A* transcripts lacking the last 32 bp of exon 3 (Δ32 bp; lower electropherogram).

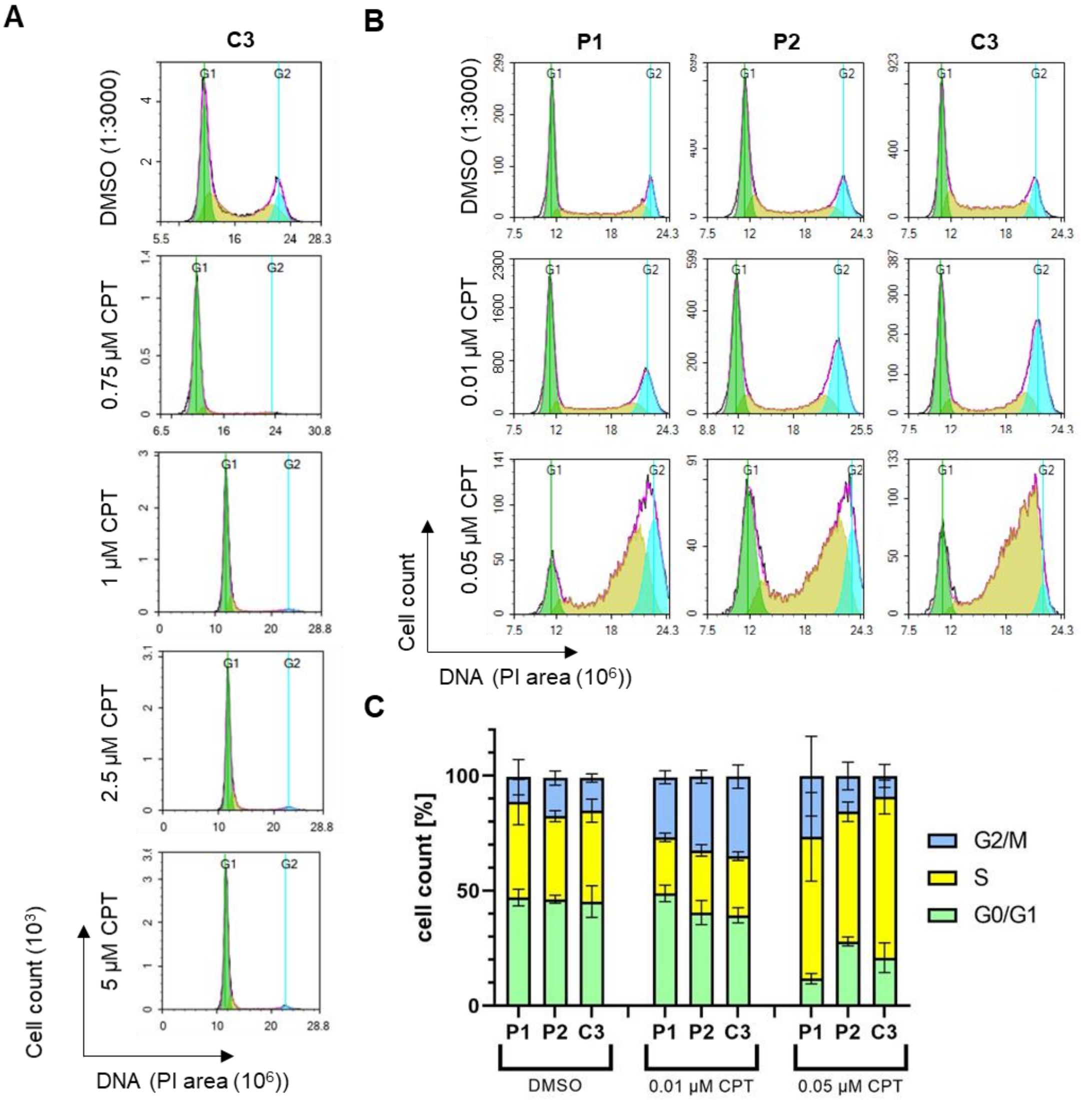

D

		DMSO	0.01 µM CPT	0.05 µM CPT
G0/ G1	P1	n.s.	**	n.s.
	P2	n.s.	n.s.	n.s.
S	P1	n.s.	n.s.	n.s.
	P2	n.s.	n.s.	n.s.
G2/ M	P1	n.s.	*	n.s.
	P2	n.s.	n.s.	n.s.

Fig. S5: Treatment of patient- and control-derived fibroblasts with the topoisomerase inhibitor camptothecin (CTP) resulted in a similar inhibition of cell cycle progression. **A, B** Patient- and control-derived fibroblasts were treated with 0.01 µM, 0.05 µM, 0.75 µM, 1 µM, 2.5 µM, and 5 µM CPT or the corresponding volume of DMSO for 24 h. The DNA content of the cells was measured by flow cytometry after propidium iodide (PI) staining. Representative histograms of the flow cytometry analysis are shown. The NovoExpress software was employed to categorize cells as G0/G1 (green peak), S (yellow peak), or G2/M (light blue peak) phase cells according to the DNA content (PI staining). **C** Quantification of patient- and control-derived fibroblasts in G0/G1, S, and G2/M phase. The bars and errors represent the mean ± SD of three independent experiments (n = 3). **D** Two-way ANOVA followed by Dunnett's *post hoc* test was used for statistical analysis separately for each cell cycle phase to compare the proportion of cells in a specific cell cycle phase between the patients and control 3. *$p \leq 0.05$; **$p \leq 0.01$. C3, control 3; n.s., not significant; P1, patient 1; P2, patient 2.

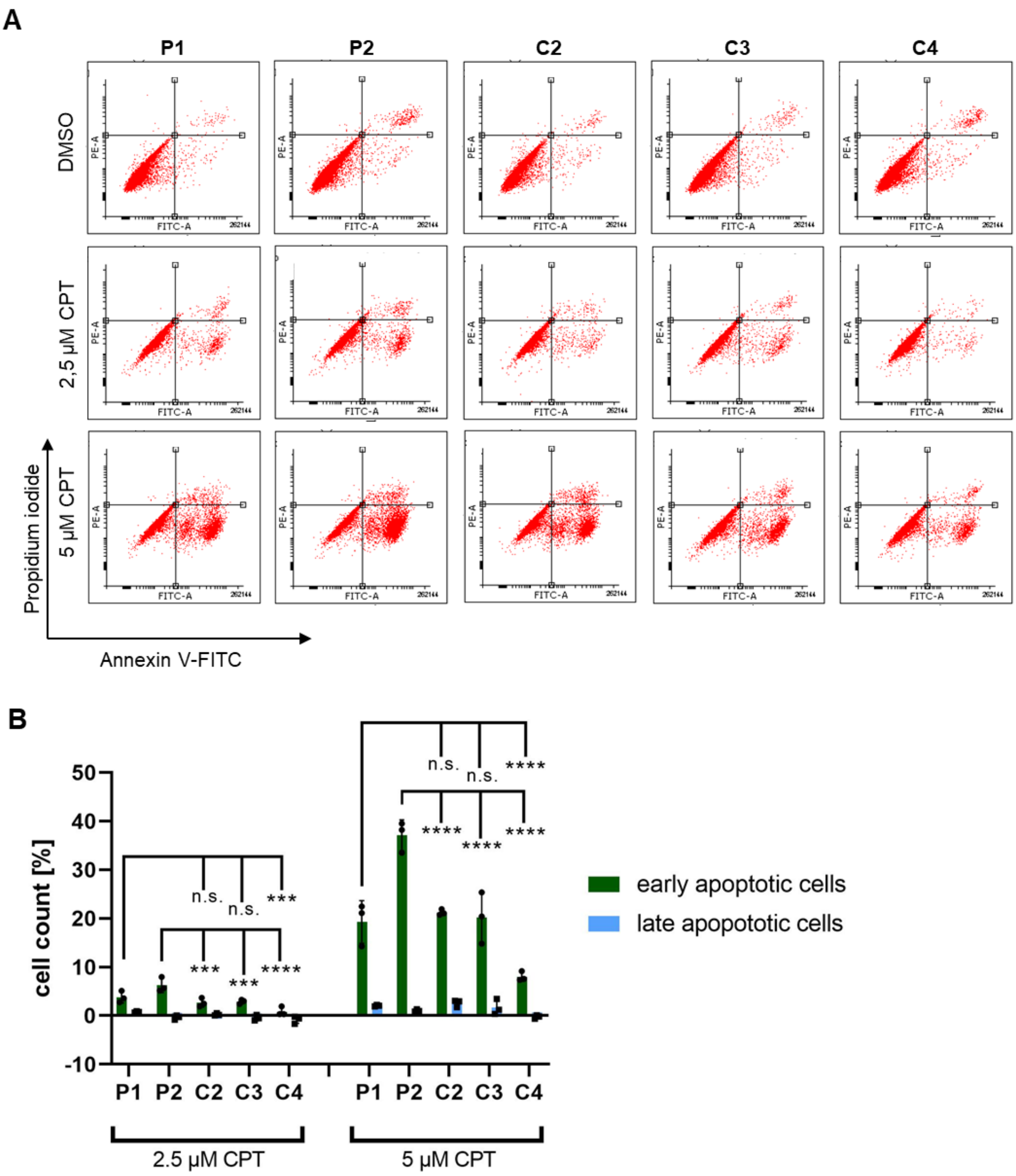

Fig. S6: Treatment of patient- and control derived fibroblasts with camptothecin (CTP) induces apoptosis. **A** Fibroblasts were treated with 2.5 µM or 5 µM CPT or with the respective amount of DMSO for 72 h. Early and late apoptotic cells were measured by flow cytometric analysis after double staining with FITC-conjugated Annexin V and propidium iodide (PI). Representative flow cytometry dot plots of the analysis are shown. The four quadrants represent normal cells in the lower left quadrant, early apoptotic cells in the lower right, late apoptotic cells in the upper right, and necrotic cells in the upper left. **B** The proportion of apoptotic cells in untreated samples was subtracted from the proportion of apoptotic cells in treated samples. The bars and errors represent the mean ± SD of three independent experiments (n = 3). Individual data points are shown. Two-way ANOVA followed by Dunnett's *post hoc* test was used for statistical analysis for 2.5 µM and 5 µM CPT to compare the proportion of early and late apoptotic cells separately between patients 1 and 2 and controls. ***$p \leq 0.001$; ****$p \leq 0.0001$. C2-C4, controls 2-4; FITC, fluorescein isothiocyanate; n.s., not significant; P1, patient 1; P2, patient 2.

SUPPLEMENTARY TABLES

Table S1. *In silico* pathogenicity and splice site predictions, minor allele frequency, and associated phenotypes (according to OMIM) for shared biallelic variants in patients 1 and 2.

Chr.	Genomic position (hg38)	Gene	mRNA reference number (MANE Select)	Nucleotide change	Amino acid alteration	Zygosity	gnomAD (v4.1.0) MAF [%]	CADD (≥20)	REVEL (≥0.6)	Alpha Missense (≥0.564)	SpliceAI (≥0.2)	OMIM phenotype (MIM number)
1	26 345 065	*CRYBG2*	NM_001039775.4	c.1593C>T	p.Val531=	hom.	0.003922	0.06	n. a.	n. a.	no impact	n. a.
5	119 197 801	*DMXL1*	NM_001290321.3	c.7590T>C	p.Leu2530=	hom.	0.005204	5.13	n. a.	n. a.	AL: 0.02	n. a.
5	150 649 601	*SYNPO*	NM_007286.6	c.1326C>T	p.Pro442=	hom.	0.003044	0.037	n. a.	n. a.	no impact	n. a.
7	51 028 008	*COBL*	NM_015198.5	c.3088C>G	p.(Gln1030Glu)	hom.	0.0002495	9.449	0.066	0.070	no impact	n. a.
11	44 075 554	*ACCS*	NM_032592.4	c.518T>C	p.(Leu173Pro)	hom.	0.008239	27.4	0.459	0.777	AL: 0.02	n. a.
11	47 178 457	*PACSIN3*	NM_016223.5	c.1068A>C	p.(Glu356Asp)	hom.	0.004957	6.747	0.06	0.070	no impact	n. a.
11	59 148 953	*FAM111A*	NM_001312909.2	c.81G>A	p.Pro27=	hom.	0.0008719	22.7	n. a.	n. a.	DL: 0.43	Kenny-Caffey syndrome, type 2 (MIM 127000; AD); Gracile bone dysplasia (MIM 602361, AD)
11	60 208 690	*MS4A4E*	NM_001393391.1	c.386C>T	p.(Thr129Met)	hom.	0.01143	5.089	n. a.	0.075	AL: 0.08	n. a.
14	45 242 387	*MIS18BP1*	NM_018353.5	c.790A>G	p.(Lys264Glu)	hom.	0.0008055	9.414	0.039	0.089	DG: 0.03	n. a.
17	74 924 835	*OTOP2*	NM_178160.3	c.203C>T	p.(Ala68Val)	hom.	0.02068	15.39	0.082	0.085	DG: 0.01	n. a.
17	74 946 831	*OTOP3*	NM_001272005.2	c.922C>T	p.(His308Tyr)	hom.	0.001117	20.6	0.077	0.079	DG: 0.01	n. a.

The functional impact of shared biallelic variants with a MAF ≤0.1% was predicted by the Combined Annotation Dependent Depletion (CADD) tool, the Rare Exome Variant Ensemble Learner (REVEL) scoring system, and the deep learning model AlphaMissense. CADD is a framework that integrates multiple annotations in one metric by contrasting variants that survived natural selection with simulated mutations. Reported CADD scores are phred-like rank scores based on the rank of that variant's score among all possible single nucleotide variants of hg19, with 10 corresponding to the top 10%, 20 at the top 1%, and 30 at the top 0.1%. The larger the score the more likely the variant has deleterious effects; the score range observed here is strongly supportive of pathogenicity, with all observed variants ranking above ~99% of all variants in a typical genome and scoring similarly to variants reported in ClinVar as pathogenic (~85% of which score >15) (7). REVEL is an ensemble method predicting the pathogenicity of missense variants with a strength for distinguishing pathogenic from rare neutral variants with a score ranging from 0-1, with a pathogenicity threshold of ≥0.6 (8). AlphaMissense is a computational tool predicting missense variant pathogenicity by combining AlphaFold-based structural information and evolutionary conservation. The score ranges from 0-1, with a pathogenicity threshold of ≥0.564 (9). Possible effect of variants on splicing was analyzed using the open-source deep learning splicing prediction algorithm SpliceAI. The score ranges from 0-1, with a cutoff of ≥0.2 (10). AD, autosomal dominant; AL, splice acceptor loss; Chr., chromosome; DG, splice donor gain; DL, splice donor loss; hom., homozygous; MAF, minor allele frequency; MANE, matched annotation between NCBI (RefSeq) and EBI (Ensembl/GENCODE); n. a., not applicable.

Table S2. Sequence of oligonucleotides used in this work.

<table>
<tr><th colspan="5">FAM111A primer sequences for variant validation and segregation analysis</th></tr>
<tr><th>Template</th><th colspan="2">Exon</th><th>Direction</th><th>Sequence (5’ → 3’)</th></tr>
<tr><td rowspan="2">DNA</td><td colspan="2" rowspan="2">3</td><td>forward</td><td>AAT GAC AGC TTT GAA GAT ACT GC</td></tr>
<tr><td>reverse</td><td>TAT AAA ACC CAA TGC CAA GG</td></tr>
<tr><th colspan="5">FAM111A primer sequences for transcript analysis (RT-PCR)</th></tr>
<tr><th>Template</th><th>Exon</th><th>Name</th><th>Direction</th><th>Sequence (5’ → 3’)</th></tr>
<tr><td rowspan="3">cDNA</td><td>2</td><td>F1</td><td>forward</td><td>GTT CTC TGC TGT TTA AAA AGT ATC C</td></tr>
<tr><td>3</td><td>F2</td><td>forward</td><td>CTG TAA GAA GCA GAG GTC ACG</td></tr>
<tr><td>4</td><td>R1</td><td>reverse</td><td>GCC TGG AGA GTG TTG AGA GC</td></tr>
<tr><th colspan="5">Primer sequences for colony PCR</th></tr>
<tr><th>Template</th><th colspan="2">Name</th><th>Direction</th><th>Sequence (5’ → 3’)</th></tr>
<tr><td rowspan="2">pCR2.1 TOPO TA cloning vector</td><td colspan="2">T7cDNA</td><td>forward</td><td>CCC TAT AGT GAG TCG TAT TA</td></tr>
<tr><td colspan="2">M13rev</td><td>reverse</td><td>CAG GAA ACA GCT ATG A</td></tr>
<tr><th colspan="5">FAM111A primer sequences for RT-qPCR</th></tr>
<tr><th>Template</th><th>Exon</th><th>Name</th><th>Direction</th><th>Sequence (5’ → 3’)</th></tr>
<tr><td rowspan="3">cDNA</td><td>2</td><td>F1</td><td>forward</td><td>GTT CTC TGC TGT TTA AAA AGT ATC C</td></tr>
<tr><td>3</td><td>Fq</td><td>forward</td><td>CAT CCG TTC ATC TTC AAG CCA TC</td></tr>
<tr><td>4</td><td>Rq</td><td>reverse</td><td>GGT CTC CTC TAG ACT CCA TCC TC</td></tr>
</table>

Exon numbering is given according to *FAM111A* reference sequence NM_022074.4.

Table S3. Laboratory measurements in patients 1 and 2.

	**Patient 1 (6 y 10 m)**	**Patient 2 (3 y 2 m)**
**Hematologic parameters**		
Hemoglobin (g/dl)	**7.2 (11.5-15.5)**	**9.4 (11.5-13.5)**
Hematocrit (PCV) (%)	**34.1 (35.0-45.0)**	**26.8 (34.0-40.0)**
Ferritin (ng/L)	**150.9 (7-140)**	n. a.
Red cell count (million/cmm)	**3.98 (4.0-5.2)**	**3.25 (3.90-5.30)**
MCV (fl)	85.7 (77.0-95.0)	82.5 (75.0-87.0)
MCH (pg)	26.9 (25.0-33.0)	28.9 (24.0-30.0)
MCHC (g/dl)	31.4 (31.0-37.0)	35.1 (31.0-37.0)
RDW (%)	**17.6 (11.0-14.0)**	**20.2 (11.0-14.0)**
Platelet count (/cmm)	**719.000 (170.000-450.000)**	**600.000 (200.000-490.000)**
D-dimer (ng/mL)	**2861 (up to 250)**	n. a.
Total leucocyte count (/cmm)	10.250	8.410 (6.000-17.000)
Basophils (/cmm)	103 (up to 100)	84 (up to 100)
Eosinophils (/cmm)	105 (up to 1000)	252 (up to 1000)
Total neutrophils (/cmm)	4305 (1500-8000)	5635 (1500-8500)
Lymphocytes (/cmm)	4920 (1500-6800)	2018 (2000-8000)
Monocytes (/cmm)	718 (200-1000)	412 (200-1000)
**Bone metabolism parameters**		
Parathyroid hormone (pg/mL)	21.1 (15.0-65.0)	28.7 (15.0-65.0)
Calcitonin (pg/mL)	2.4 (<5.0)	1.3 (<12)
Serum ionized Calcium (mmol/L)	n. a.	1.33 (1.1-1.35)
Serum calcium (mg/dL)	n. a.	9.7 (8.8-10.8)
Serum inorganic phosphorus (mg/dL)	n. a.	6.43 (4-7)
Alkaline Phosphatase (U/L)	n. a.	198 (up to 290)
Basal growth hormone (ng/mL)	**4.27 (>7)**	n. a.
**Hepatic parameters**		
ALT (SGPT) (U/L)	24 (up to 33)	28 (up to 41)
AST (SGOT)	**47 (up to 32)**	n. a.
LDH (U/L)	281 (up to 615)	n. a.

Age-matched reference values are given in brackets. Patient values that differ from the reference values are highlighted in bold. ALT, alanine aminotransferase; AST, aspartate aminotransferase; LDH, lactate dehydrogenase; m, months; MCV, mean corpuscular volume; MCH, mean corpuscular hemoglobin; MCHC, MCH concentration; n. a., not available; PCV, packed cell volume; RDW, red cell distribution width; SGOT, serum glutamate-oxaloacetate transaminase; SGPT, serum glutamic-pyruvic transaminase; y, years.

Table S4. Top 10 Phenomizer disease associations using short stature, thickened cortex of long bones, and stenosis of the medullary cavity of the long bones as search keys.

***p* value**	**Score**	**Disease entry according to Phenomizer**	**Disease entry according to the "*Nosology of genetic skeletal disorders: 2023 revision*" (11)**	**Disease gene(s)**
0.0320	5.6001	Kenny-Caffey syndrome, type 2 (MIM 127000)	Kenny-Caffey syndrome, dominant, *FAM111A*-related (NOS 21-0050; AD)	*FAM111A*
1.0000	3.9233	Stüve-Wiedemann syndrome/Schwartz-Jampel type 2 syndrome (MIM 601559)	Stüve-Wiedemann syndrome, *LIFR*-related (NOS 20-0020; AR)	*LIFR*
1.0000	3.9233	Craniofacial dysostosis with diaphyseal hyperplasia	–	–
1.0000	3.8986	Camurati-Engelmann disease (MIM 131300)	Diaphyseal dysplasia Camurati-Engelmann, *TGFB1*-related (NOS 25-0140; AD)	*TGFB1*
1.0000	3.8489	Schinzel-Giedion midface retraction syndrome (MIM 269150)	–	*SETBP1*
1.0000	3.8254	Diaphyseal medullary stenosis with malignant fibrous histiocytoma (MIM 112250)	Diaphyseal medullary stenosis with malignant fibrous histiocytoma, *MTAP*-related (NOS 25-0330; AD)	*MTAP*
1.0000	3.7422	Endosteal hyperostosis (MIM 144750)	Endosteal hyperostosis, Worth type (NOS 25-0290; AD)	*LRP5*
1.0000	3.6106	Sclerosteosis 1 (MIM 269500); Sclerosteosis 2 (MIM 614305)	Sclerosteosis, *SOST*-related (NOS 25-0260; AR); Sclerosteosis, *LRP4*-related (NOS 25-0270; AR)	*SOST, LRP4*
1.0000	3.3431	Lenz-Majewski hyperostotic dwarfism (MIM 151050)	Lenz-Majewski hyperostotic dysplasia, *PTDSS1*-related (NOS 25-0360; AD)	*PTDSS1*
1.0000	3.3431	Grant syndrome (MIM 138930)	–	–

The Phenomizer (https://compbio.charite.de/phenomizer/) (12, 13) was used with the HPO terms short stature (HP:0004322), thickened cortex of long bones (HP:0000935), and stenosis of the medullary cavity of the long bones (HP:0100254) as search terms (last accessed 07/2024). Top 10 disease associations are listed. –, no data; AD, autosomal dominant; AR, autosomal recessive.

Table S5. *In silico* splice site predictions for the *FAM111A* c.81G>A variant.

	**wild-type sequence**	**mutated sequence**
**SpliceSiteFinder-like (range 0-100)**	80.9	–
**NNSPLICE (range 0-1)**	1.0	0.6
**MaxEntScan (range 0-16)**	9.2	5.8
**GeneSplicer (range 0-21)**	3.1	–

Donor and acceptor splice site prediction scores were calculated for wild-type and mutated sequences using Alamut Visual v2.15 (SOPHiA GENETICS, Lausanne, Switzerland) which implements the following splice site prediction algorithms: SpliceSiteFinder (14, 15), NNSPLICE 0.9 version (16), MaxEntScan (17), and GeneSplicer (18). High and low scores indicate strong and weak splice sites, respectively. –: splice site not recognized.

Supplementary references

1. Chen S, Zhou Y, Chen Y, Gu J. fastp: an ultra-fast all-in-one FASTQ preprocessor. Bioinformatics. 2018;34(17):i884-i90.
2. Li H, Durbin R. Fast and accurate long-read alignment with Burrows-Wheeler transform. Bioinformatics. 2010;26(5):589-95.
3. Kim S, Scheffler K, Halpern AL, Bekritsky MA, Noh E, Kallberg M, et al. Strelka2: fast and accurate calling of germline and somatic variants. Nat Methods. 2018;15(8):591-4.
4. McKenna A, Hanna M, Banks E, Sivachenko A, Cibulskis K, Kernytsky A, et al. The Genome Analysis Toolkit: a MapReduce framework for analyzing next-generation DNA sequencing data. Genome research. 2010;20(9):1297-303.
5. McLaren W, Gil L, Hunt SE, Riat HS, Ritchie GR, Thormann A, et al. The Ensembl Variant Effect Predictor. Genome Biol. 2016;17(1):122.
6. Eren E, Tezcan Unlu H, Ceylaner S, Tarim O. Compound Heterozygous Variants in FAM111A Cause Autosomal Recessive Kenny-Caffey Syndrome Type 2. J Clin Res Pediatr Endocrinol. 2021;15(1):97–102.
7. Kircher M, Witten DM, Jain P, O'Roak BJ, Cooper GM, Shendure J. A general framework for estimating the relative pathogenicity of human genetic variants. Nat Genet. 2014;46(3):310-5.
8. Ioannidis NM, Rothstein JH, Pejaver V, Middha S, McDonnell SK, Baheti S, et al. REVEL: An Ensemble Method for Predicting the Pathogenicity of Rare Missense Variants. American Journal of Human Genetics. 2016;99(4):877-85.
9. Cheng J, Novati G, Pan J, Bycroft C, Zemgulyte A, Applebaum T, et al. Accurate proteome-wide missense variant effect prediction with AlphaMissense. Science. 2023;381(6664):eadg7492.
10. Jaganathan K, Kyriazopoulou Panagiotopoulou S, McRae JF, Darbandi SF, Knowles D, Li YI, et al. Predicting Splicing from Primary Sequence with Deep Learning. Cell. 2019;176(3):535-48 e24.
11. Unger S, Ferreira CR, Mortier GR, Ali H, Bertola DR, Calder A, et al. Nosology of genetic skeletal disorders: 2023 revision. Am J Med Genet A. 2023;191(5):1164-209.
12. Kohler S, Schulz MH, Krawitz P, Bauer S, Dolken S, Ott CE, et al. Clinical diagnostics in human genetics with semantic similarity searches in ontologies. Am J Hum Genet. 2009;85(4):457-64.
13. Kohler S, Vasilevsky NA, Engelstad M, Foster E, McMurry J, Ayme S, et al. The Human Phenotype Ontology in 2017. Nucleic Acids Res. 2017;45(D1):D865-D76.
14. Zhang MQ. Statistical features of human exons and their flanking regions. Hum Mol Genet. 1998;7(5):919-32.
15. Shapiro MB, Senapathy P. RNA splice junctions of different classes of eukaryotes: sequence statistics and functional implications in gene expression. Nucleic Acids Res. 1987;15(17):7155-74.
16. Reese MG, Eeckman FH, Kulp D, Haussler D. Improved splice site detection in Genie. J Comput Biol. 1997;4(3):311-23.
17. Yeo G, Burge CB. Maximum entropy modeling of short sequence motifs with applications to RNA splicing signals. J Comput Biol. 2004;11(2-3):377-94.
18. Pertea M, Lin X, Salzberg SL. GeneSplicer: a new computational method for splice site prediction. Nucleic Acids Res. 2001;29(5):1185-90.
